# Genetic characterization of nodular worm infections in Asian Apes

**DOI:** 10.1038/s41598-021-86518-2

**Published:** 2021-03-31

**Authors:** Erhan Yalcindag, Peter Stuart, Hideo Hasegawa, Adrian Streit, Jana Doležalová, Helen Morrogh-Bernard, Susan M. Cheyne, Wisnu Nurcahyo, Ivona Foitová

**Affiliations:** 1 Department of Botany and Zoology, Faculty of Science, Masaryk University, Kotlářská 2, 611 37 Brno, Czech Republic; 2 The Roslin Institute, Royal (Dick) School of Veterinary Studies, University of Edinburgh, Easter Bush, Midlothian, EH25 9RG UK; 3 Department of Biological and Pharmaceutical Sciences, Munster Technological University, Tralee, Co. Kerry Ireland; 4 Department of Biomedicine, Faculty of Medicine, Oita University, 1-1 Idaigaoka, Hasama, Yufu, Oita 879-5593 Japan; 5 Department Evolutionary Biology, Max Planck Institute for Developmental Biology, Max-Planck-Ring 9, 72076 Tübingen, Germany; 6 Department of Physiology, Faculty of Veterinary Medicine, University of Veterinary and Pharmaceutical Sciences, Brno, Palackého tř. 1, Brno, Czech Republic; 7 Borneo Nature Foundation, Palangkaraya, Central Kalimantan Indonesia; 8 Department of Humanities and Social Sciences, Oxford Brookes University, Oxford, UK; 9 Department of Parasitology, Faculty of Veterinary Medicine, Gadjah Mada University, Yogyakarta, Indonesia

**Keywords:** Evolution, Molecular biology

## Abstract

Parasitic nematodes of *Oesophagostomum* spp., commonly known, as 'nodular worms' are emerging as the most widely distributed and prevalent zoonotic nematodes. *Oesophagostomum* infections are well documented in African non-human primates; however, the taxonomy, distribution and transmission of *Oesophagostomum* in Asian non-human primates are not adequately studied. To better understand which *Oesophagostomum* species infect Asian non-human primates and determine their phylogeny we analysed 55 faecal samples from 50 orangutan and 5 gibbon individuals from Borneo and Sumatra. Both microscopy and molecular results revealed that semi-wild animals had higher *Oesophagostomum* infection prevalence than free ranging animals. Based on sequence genotyping analysis targeting the Internal transcribed spacer 2 of rDNA, we report for the first time the presence of *O. aculeatum* in Sumatran apes. Population genetic analysis shows that there is significant genetic differentiation between Bornean and Sumatran *O. aculeatum* populations. Our results clearly reveal that *O. aculeatum* in free-ranging animals have a higher genetic variation than those in semi-wild animals, demonstrating that *O. aculeatum* is circulating naturally in wildlife and zoonotic transmission is possible. Further studies should be conducted to better understand the epidemiology and dynamics of *Oesophagostomum* transmission between humans, non-human primates and other wild species and livestock in Southeast Asia.

## Introduction

Orangutans and gibbons are the only ape species living in Southeast Asia and both are primarily arboreal species. Orangutans show a 97% genetic similarity with humans^1^. Today, orangutans are divided into three species: the Bornean orangutans (*Pongo pygmaeus*), the Sumatran orangutans (*Pongo abelii*) and *Pongo tapanuliensis,* which has recently been separated from *P. abelii*^2^. Gibbons are smaller apes belonging to the family Hylobatidae with four recognized genera: *Hoolock*, *Nomascus*, *Hylobates* and *Symphalangus*^3^. Overall, 20 gibbon species have been identified, and only *Hylobates* are reported in both Sumatra and Borneo whilst *Symphalangus* are only found in Sumatra and South Malaysia^4^. The Asian apes are currently listed by the IUCN as Critically Endangered^5^. These species are severely threatened by poaching, habitat conversion, destruction through deforestation and other human activities. These threats may increase the opportunities for contact with other wildlife species, livestock and humans resulting in increased disease transmission between them. Infectious diseases, possibly linked to environmental changes, may play a role in population declines through increased mortality and morbidity of these species^6^. In addition, the zoonotic potential of pathogens in non-human primates (hereafter referred to as NHPs) has received considerable attention due to increased contact between domestic communities and local primate species^7^. While several parasitic and viral infections are well documented in African apes, the distribution and transmission of parasites of Asian apes is still relatively neglected and or poorly understood^8^. However, in the last decades, new pathogens have been discovered in Asian apes^9–13^ as well as new studies of previously known ones^6,8,14–18^. The health of the NHPs are affected by a large variety of soil-transmitted helminths (STHs), some with zoonotic potential, e.g. roundworms (*Ascaris lumbricoides*), whipworms (*Trichuris trichiura*), hookworms (*Necator americanus, Ancylostoma duodenale*), threadworms (*Strongyloides stercoralis*) and also nodular worms (*Oesophagostomum* spp.)^19–25^. Increased contact with humans has been identified as a risk factor of infection, making primates who have undergone human rehabilitation (semi wild) more likely to be infected than wild individuals^12^.

*Oesophagostomum* species are stout, white roundworms and have a direct life cycle. Eggs passed in the faeces, hatch and rapidly develop into L1 rhabditiform larvae. After 24 h of hatching, L2 become infective L3 within 3–4 days. L3 keep the protective cuticle of L2 and are capable of surviving long periods of adverse environmental conditions, e.g., hot-dry conditions of the dry season. Infection occurs by ingestion of filariform L3 larvae via vegetation eaten by the host. After ingestion, L3 pass to the cecum, where they exsheath within ~ 3 days of ingestion then invade the tunica mucosa, stimulating the formation of separate cysts around individual larvae in the gut wall. The larvae develop there to the L4 stage then once in the lumen, the larvae moult and reach the adult stage^26^.

For most nematodes parasites, species identification based on egg morphology is difficult, therefore recently molecular techniques are widely used to identify species. Accordingly, recent molecular genetic studies have demonstrated that the ITS nucleotide sequence of rDNA allows an unequivocal identification and discrimination of a range of strongylid nematode species, irrespective of the developmental stage of the parasites^27^.

Based on molecular findings*, Oesophagostomum* spp. are known to frequently infect domestic and wild pigs, ruminants and primates^28–31^. Eight species of *Oesophagostomum* have been observed to occur in African NHPs and three of them (*O. bifurcum*, *O. stephanostomum* and *O. aculeatum*) are also reported in humans^32–34^. Human cases have been attributed to an animal origin and NHPs have been proposed as a potential reservoir^33^. Infections in wild primates appear to be asymptomatic, although clinical signs and mortality due to *Oesophagostomum* have been recorded in captive settings^35^. *Oesphagostomum* spp. have been reported from orangutans based on morphological and coprological analysis^36^. Arizono et al.^37^ molecularly identified *O. aculeatum* infections for the first time in Japanese macaques. Recently, molecular markers showed a widespread distribution of *O. aculeatum* in the Bornean primate community, including orangutans^38^, although there is a paucity of investigations in gibbons. Currently, there is no study done on *O. aculeatum* distribution in the Sumatran NHPs community. The aim of this study was therefore to genetically investigate *Oesphagostomum* parasitising in wild and semi-wild Asian apes (two orangutan species; *P. pygmaeus* and *P. abelii* and one gibbon species; *Hylobates albibarbis*) to confirm for the first time if Sumatran orangutans and wild Asian gibbons are being infected and to investigate the distribution and population structure of nodular worm infections in these species.

## Results

**Figure 1:**
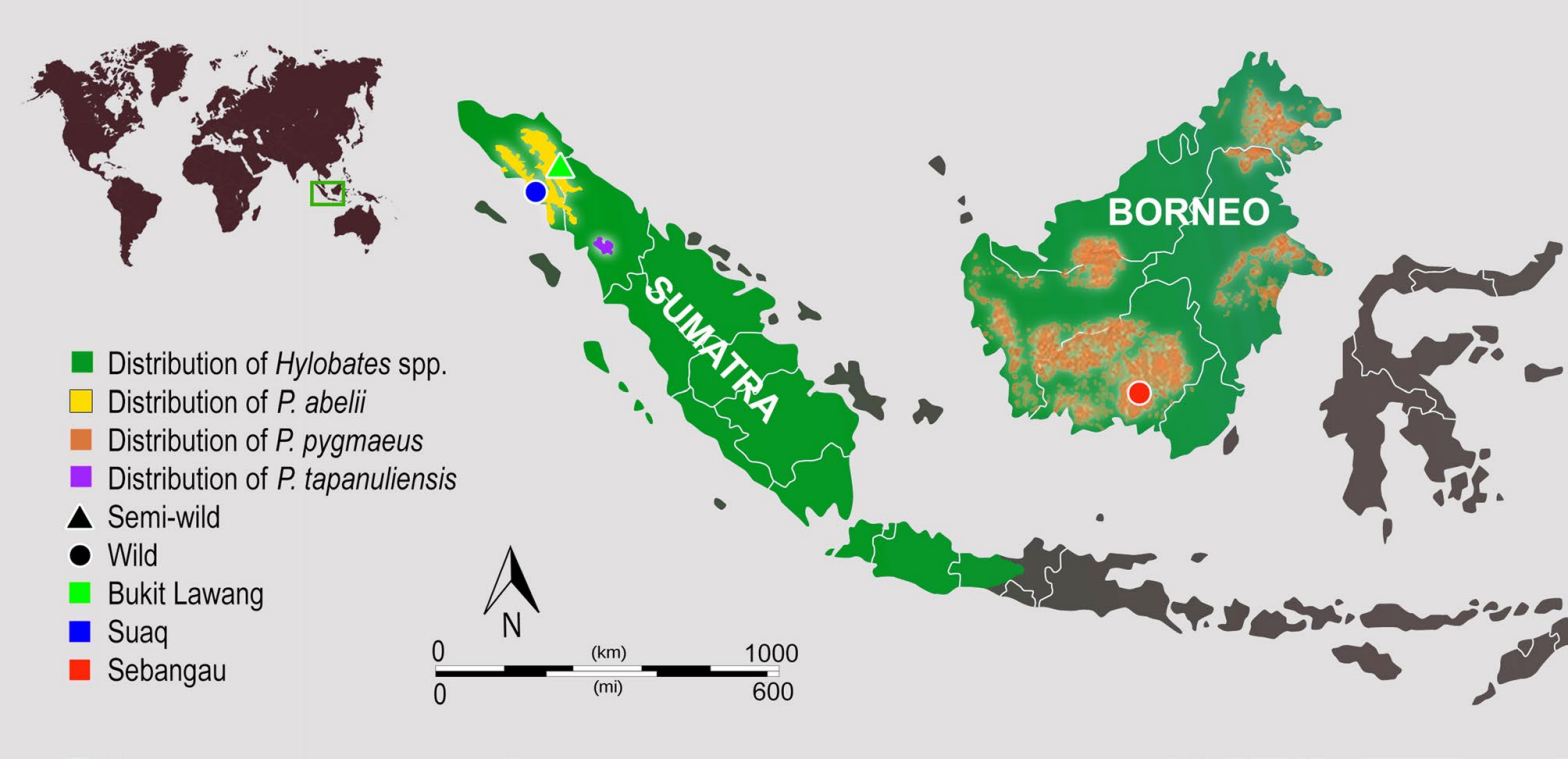
Geographical location of study sites in Borneo and Sumatra. For more information, please refer to Table 1 and Supporting Table S1. The map background was downloaded from free maps platform system (http://www.freepik.com) and modified in Adobe Photoshop CS6.

A total of 55 faecal samples from 50 different orangutans and 5 from different gibbon individuals were collected from three different site. (Fig. 1, Table 1 and Supp Table 1). From these, 35 orangutans and 3 gibbons appeared to have *Oesophagostomum* and/or hookworm nematode-like eggs by microscopy, population-wide prevalence of infection 69% (ranging 52.6%—90% (Table 1). All eggs identified by microscopy as *Oesophagostomum* were similar in internal and external morphology in samples from all primate species. The size of eggs was 60–85 by 35–50 μm, similar to previous records^28,39^. For each population, the mean size of the eggs measured was calculated and no significant difference between populations was observed (chi-square = 0.063, df = 2, p-value > 0. 05) (Supp Table 3).

**Table 1:** Number of Asian ape’s fecal samples collected and sequenced in this study with site, populations, forest type, geographical coordinates, total sample size, and number of infected samples.

Site	Host species	Population	Short Name	Forest type	Density	Latitude Longitude	Sample size	Positive samples	Prevalence (%95)	Males (n)	Females (n)
Borneo^†^	*Pongo pygmaeus*	Sebangau	Seb	Peat swamp	2.3 ind/km^2^ ^73^	2° 07′S 112°22′E	11	6^a^/3^b^	54.55^a^ (28–79) 27.27^b^ (10–57)	2^a^ (7^a^)	1^a^ (4^a^)
Borneo^†^	*Hylobates albibarbis*				2.59 groups/km^2^ ^74^		5	3^a^/3^b^	60^a^ (23–88) 60^b^ (23–88)	^§^	
Sumatra^†^	*Pongo abelii*	Suaq	Suaq	Coastal swamp	7 ind/km^2^ ^75^	3° 42′N 97°26′E	19	10^a^/6^b^	52.63^a^ (32–73) 31.58^b^ (15–54)	4^a^ (13^a^)	2^a^ (6^a^)
Sumatra^‡^	*Pongo abelii*	Bukit Lawang	Buk	Hill dipterocarp	1.8 ind/km^2^ ^73^	3°47′N 98°08′E	20	18^a^/15^b^	90 ^a^ (70–97) 75^b^ (53–89)	5^a^ (7^a^)	10^a^ (13^a^)
Total							55	37^a^/27^b^	69.1^a^ (56–80) 49.1^b^ (36–62)	11^a^ (27^a^)^¥^	13^a^ (23^a^)^¥^

^a^Positive samples from microscopy.

^b^Positive samples from PCR.

^†^Populations of free ranging animals.

^‡^ Populations of semi-wild animals.

^§^3 of 6 gibbons sex were unknown, data not include in the table.

^¥^Infected orangutan samples by sex.

Polymerase Chain Reaction (here after, PCR) generated single clear amplicons from both the primer couples, of expected length for all microscopy-positive individuals. Despite PCR positive *Oesophagostomum* infection, with a prevalence of 49.1% [95% CI; 36.3%–61.9%], the microscopy prevalence was 69.1% [95% CI; 55.9%–79.7%] (Table 1). 11 positive samples from microscopy were negative by PCR.

Based on PCR positive results, no significant difference was found in the frequency of infection between the wild populations of the two different species (*P. abelii* 31.6%, N = 19, *P. pygmaeus* 27.3%, N = 11; χ2 = 0.062, 1 df; p = 0.8041), as well as between all Sumatran and Bornean orangutan samples (all *P. abelii* 53.8%, N = 39, *P. pygmaeus*; 27.8%, N = 11; χ2 = 1.479, 1 df; p = 0.2239). Semi‐wild orangutans had significantly higher frequencies of infection with *O. aculeatum* than wild orangutans (semi‐wild 75%, N = 20, wild 30%, N = 30; χ2 = 8.016, 1 df; p = 0.0046). The same patterns observed between only *P. abelii* semi-wild and wild individuals (semi-wild *P. abelii* 75%, N = 20, wild *P. abelii* 31.57%, N = 19; χ2 = 5.748, 1 df; p = 0.0165). No significant association was detected between orangutan sex and frequency of infection with *O. aculeatum* (female 56.5%, N = 23, male 40.7%, N = 27; χ2 = 0.688, 1 df; p = 0.407). Regarding the wild male and wild female individuals, no significant difference was found (wild male 30%, N = 20, wild female 30%, N = 10; χ2 = 0. 1 df; p = 1). The same patterns was also observed in both semi-wild both female and male orangutans (semi-wild female 76.4%, N = 13, semi-wild male 71.4%, N = 7; χ2 = 0.073, 1 df; p = 0.7866).

However, we did not find any significant difference in the frequency of *O. aculatum* infection within the two sympatric different host species; (*P. pygmaeus* 27.3%, N = 11, *H. albibarbis* 60%, N = 5; χ2 = 0.485, 1 df; p = 0.4862) or between all orangutans population versus gibbons (all orangutans 48%, N = 50, gibbons 60%, N = 5; χ2 = 0.002, 1 df; p = 0.966).

**Figure 2:**
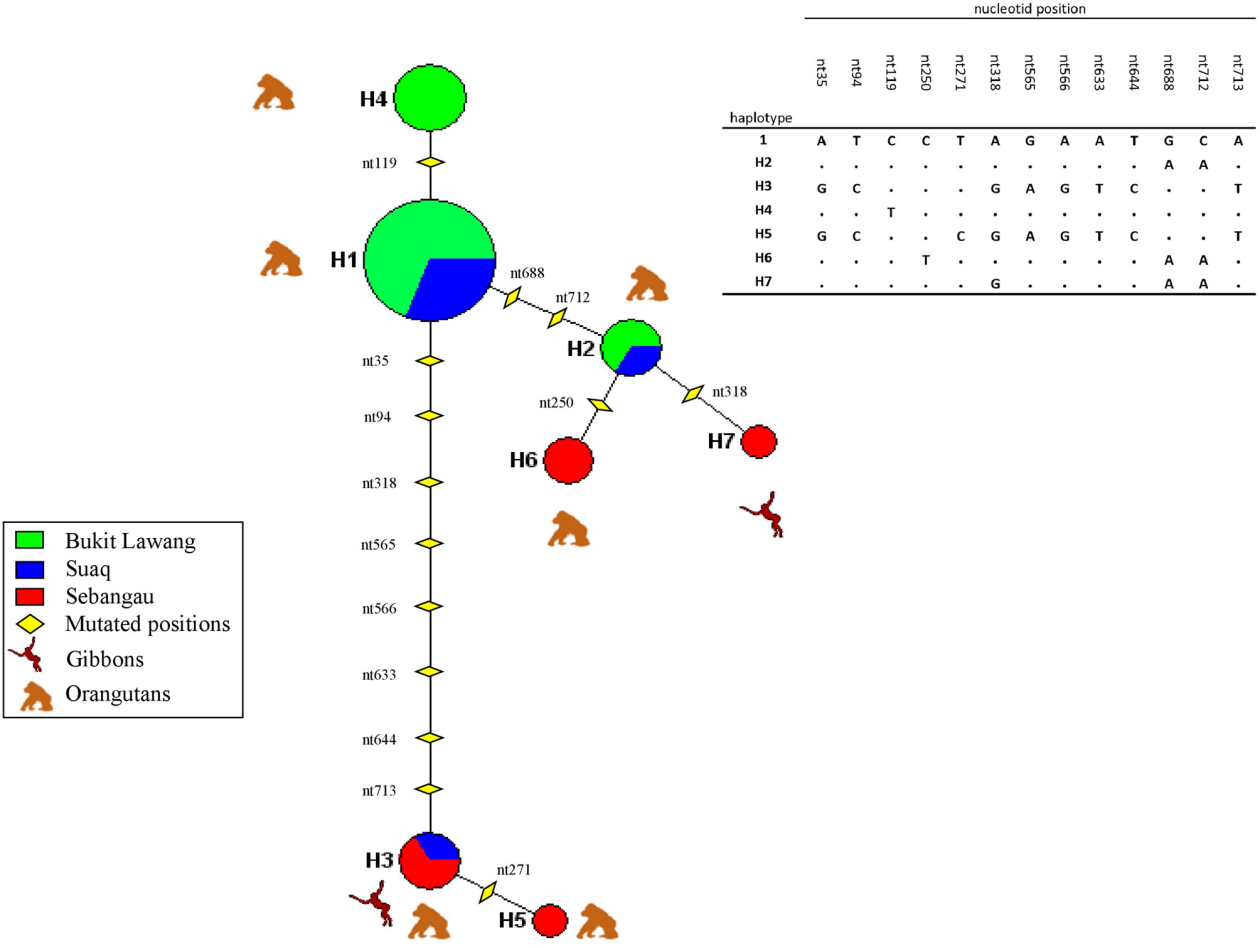
(**a**) Haplotype networks constructed using NETWORK 5.0.0.0. The network was built using a 758-bp-long fragment containing the ITS1-5.8S-ITS2 rDNA from 27 *O. aculeatum* specimen from this work. Node sizes (circles) are proportional to haplotype frequencies. Node colors indicate geographic origin. (**b**) Detected mutation positions on the 758-bp-long fragment containing the ITS1-5.8S-ITS2 rDNA.

We obtained a 758-bp-length fragment of ITS1-5.8S-ITS2 ribosomal DNA (here after, rDNA) from a total of 27 different individuals, 3 from gibbons and 24 from orangutans. By comparison with published sequences using NCBI BLAST, our samples showed a 98% identity for *O. stephanostomum* (AB821014.1), 95% *O. detantum* (AJ619979.1) and 94% *Oesophagostomum sp*. MOS-2014 (AB908964.1). The resulting long DNA sequence overlapped with published sequences and contained no insertions or deletions, making alignment unequivocal. After analysis of the 27 *Oesophagostomum* sequences, we identified seven unique haplotypes. H1, H4, H2 and H3 were the most frequent haplotype detected in 48%, 14%, 11% and 11% of the infections, respectively. There is no shared haplotype between the two wild orangutan species *P. pygmaeus;* Bornean Sebangau and *P. abelii;* Sumatran Suaq (hereafter referred to as Seb and Suaq respectively). Only two haplotypes (H1 and H2) are shared between the Sumatran populations of Suaq and the semi-wild population in Bukit Lawang (hereafter referred to as the Buk). H3 is the only haplotype shared between wild *P. pygmaeus* and *H. albibarbis* in Sumatra and H5, H6 and H7 are unique haplotypes represented only in Seb (Fig. 2a). The nucleotide position of polymorphic sites are presented in Fig. 2b.

**Figure 3:**
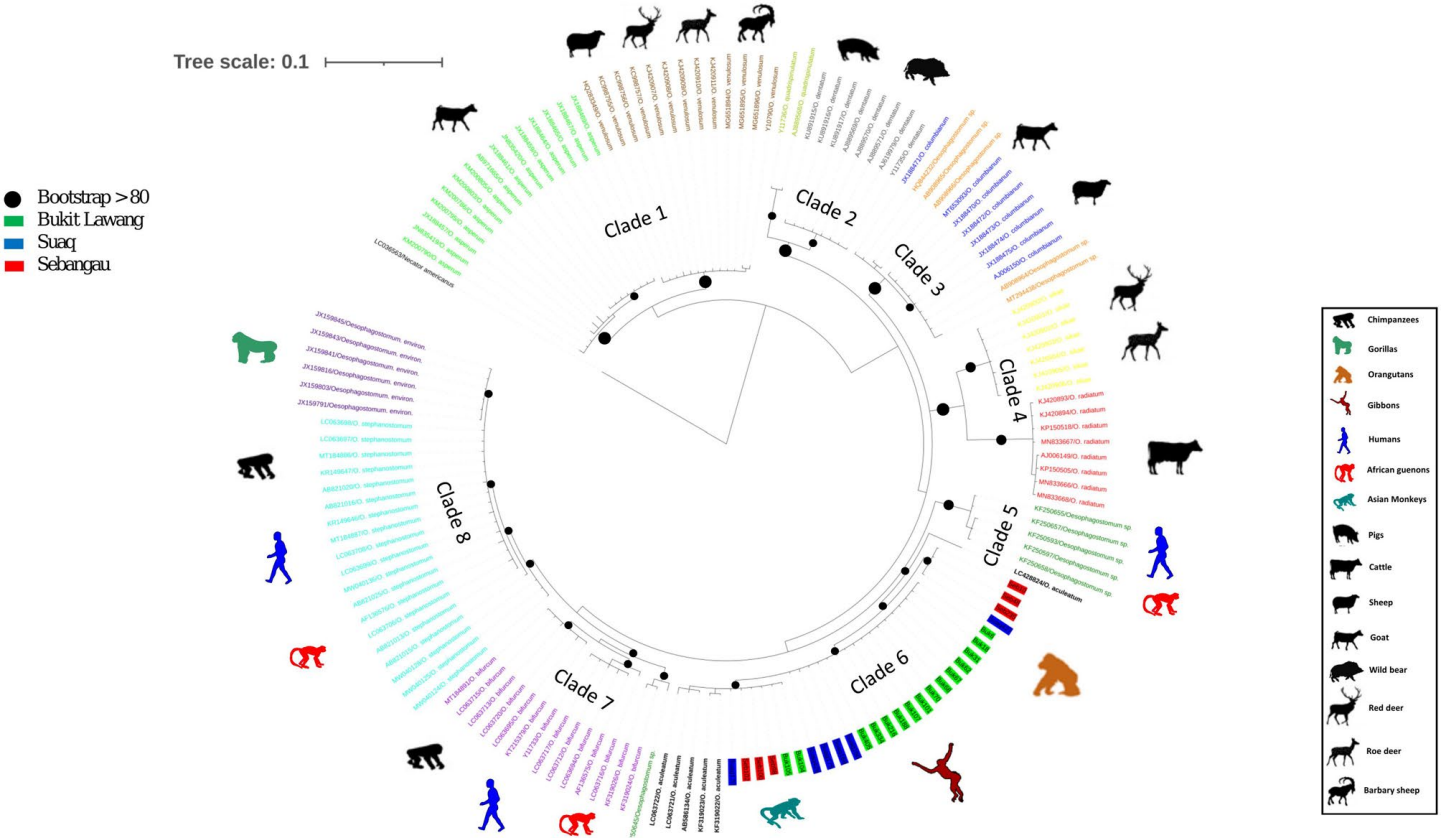
Phylogenetic relationship among *Oesophagostomum* isolates based on ITS2 rDNA (258 bp) sequences. The best-fitting model under the Akaike information criterion was HKY86 + G6 for nucleotides as identified by JModelTest v.0.1.1. Scale bar indicates nucleotide substitution per site. High (> 80) bootstrap support are indicated next to the respective node by dots in black, respectively. *O. aculeatum* sequences are identified in this study are indicated with text-highlighted colour, sequences taken from NCBI are presented with font colour.

The best sequence evolution of *Oesophagostomum* sequences obtained in this study with published other *Oesophagostomum* species sequences including outgroup, were HKY86 + G6 by JModelTest^40^. Published reference sequences were included to identify putative species and only 258 bp sequence were available. Based on phylogenetic analyses of the *Oesophagostomum* ITS2 rDNA (258 bp) sequences, the phylogenetic tree resolved these sequences into 8 clades according to host species (Fig. 3). Clade 1 composed of all *O. asperum* sequences in one group and a second distinct *O. venulosum* group that are infecting mainly bovidae species. Clade 2 is represented by two groups. The first contains both *O. quadrispinulatum* and the second contained all *O. dentatum* sequences. Clade 3 contained all *O. colombianum* samples as well as *Oesophagostomum sp.* only infecting small domestic ruminants. Clade 4 is divided into 2 groups with, one *O. sikae* isolates from deer and all *O. ruminatum* sequences isolated from cattle.

The Clade 5 contained *Oesophagostomum sp*. only infected human and African monkeys.

The clade 7 contained all sequences of *O. bifurcum* and clade 8 contained all *O. stephanostomum* sequences from different host species. Clade 6 was composed of all orangutan sequences as well as three sequences from gibbons with all *O. aculeatum* sequences with a 96–99% similarity.

Tree topologies showed a strong pattern of geographical structure across the geographic range of *Oesophagostomum* spp*.* between Southeast Asian and African primates. Our results show that Southeast Asian NHPs are infected by *O. aculeatum* only (Fig. 3). Within the clade 6, we observed two groups. The first group contained two sequences from gibbons and one sequence from Sumatran orangutan and one sequence from a Bornean orangutan. The second group contained all of the sequences from semi-captive Sumatran orangutans. Six additional sequences; one sequence from a gibbon, two sequences from two Bornean orangutans, two sequences from two semi-wild Sumatran orangutans and one sequence from one free ranging Sumatran orangutan sorted into group two which were 99% similar to the same *O. aculeatum* reference sequences.

The total number of sequences obtained and analysed for each population is given in Table 2. Overall, the Bornean (Seb) population exhibited the greatest haplotype diversity (0.867) and nucleotide diversity (0.0089). However, the Sumatran populations showed almost the same haplotype diversity in both (0.6 for Suaq and 0.59 for Buk) but the Suaq population displayed four times higher nucleotide diversity than the Buk population (Table 2).

**Table 2:** Summary statistics on polymorphism of *ITS1–5.8S–ITS2* genes in three populations of *O. aculeatum* collected from orangutans and gibbons inhabiting Borneo and Sumatra.

Population	Size (bp)	N	S	h	Hd	π	D
Suaq	758	6	10	3	0.6	0.0044	3.33
Buk	758	15	3	3	0.59	0.0012	0.914
Seb	758	6	12	4	0.867	0.0089	6.8
Total	758	27	13	7	0.741	0.0044	3.345

N, Number of Sequences obtained; S, Number of polymorphic sites; h, Number of haplotype; Hd, Haplotype diversity; π, Nucleotide diversity; D, Average number of nucleotide difference between sequences.

At host intra species level, nucleotide diversity estimated from the rDNA marker was 3.6- fold higher in Suaq (Sumatran wild) then Buk (Sumatran semi-wild). At host inter species level, Seb (Bornean wild) samples had the highest nucleotide diversity; 7.5 and 2 times higher than semi-wild and wild populations from Sumatra, respectively.

Genetic differentiation between Sumatran and Bornean populations was very strong as shown by high pairwise *Fst* estimates at rDNA (Table 3). Regarding genetic differentiation between populations, the lowest *Fst* values was observed between Buk and Suaq populations (*Fst* = 0.06882, *P* > 0.05) while the highest significant genetic differentiation was observed between Buk and Seb (*Fst* = 0.49204, P < 0.05) (Table 3). The analysis of the three different *O. aculeatum* populations showed that there is no correlation between the genetic distances with geographic distance (Supplementary Figure S1).

**Table 3:** Estimates of genetic differentiation (F_ST_) obtained between pairs of populations (in bold) and p-value of the test of differentiation (in italics) for ITS1-5.8S-ITS2 gene.

ITS1-5.8S-ITS2	Suaq	Buk	Seb
Suaq		*0.18919*	*0.15315*
Buk	**0.06882**		*0*
Seb	**0.09703**	**0.49204**	

## Discussion and conclusions

In this study, we extracted DNA directly from nematodes eggs, without coprocultures and genetic characterization of L3 larvae, to identify nodular worm infection in Asian NHPs. Based on the internal transcribed spacer 2 (ITS 2) region of ribosomal RNA gene (rDNA), we confirmed that *O. aculeatum* is infecting Asian apes in Borneo. We confirm for the first time that Sumatran NHPs are also infected with this parasitic nematode. We confirm by molecular techniques that gibbons can be infected and therefore should be included in future studies of nodular worms, such as investigating how their unique ecology effects their risk of infection.

Mitochondrial and nuclear genes are used in phylogenetic studies on strongylid species^41^. Based on the mitochondrial cox1 gene, Frias et al.^38^ showed that Bornean primates are subject to *O. aculeatum* infection. However, in our study we used ITS sequences to discriminate nematode species as the ITS regions are among the most variable nuclear loci, with a sufficient number of comparative sequences in databases. Our result demonstrated the same phylogenetic similarity observed from Frias et al.^38^. Accordingly, the rapid mode of ITS evolution including frequent indels makes alignment of ITS haplotypes more challenging compared to protein-coding markers, which can adversely affect the reliability of phylogenetic reconstruction^42^.

The evolution and dispersion of strongylid parasites and their relationships with other STHs species found in NHPs especially in hominids have been a point of attention^28,36,43^. Several studies have shown that *Oesophagostomum* infections of African primates are mainly *O. stephanostomum*, *O. bifurcum* and those belonging to the unclassified *Oesophagostomum* sp. and presented in three genetic lineages^27,28,44,45^. Later, Ota et al.^46^, showed the existence of a fourth clade, *O. aculeatum* clade from Asian primates, distinct from the three clades of African species. However, the molecular phylogenies have been limited by a deficiency of representative samples from Asian apes. In our study, based on ITS and 5.8S nucleotide sequence information, we identified *Oesophagostomum* sp. as one of the prevalent helminth parasites of Asian non-human primates. The *Oesophagostomum* sequences recovered from Asian apes clustered within the *O. aculeatum* clade. Interestingly, we did not detect any other nodular worm infections in our dataset, as it is known that the primer set used in our study are able to detect other *Oesophagostomum* species^46^, suggesting that *O. aculeatum* is the only or at least the predominant *Oesophagostomum* species that parasitizes orangutans and gibbons in our study area. Recently, Frias et al.^38^ showed that *O. aculeatum* is widely distributed in Borneo and that it infected all studied members of host species, such as long‐tailed macaques, silvered langurs, proboscis monkeys and Bornean orangutans, with little clear genetic differentiation among them.

Previous studies have indicated that captive and/or semi-wild orangutans are subject to a higher prevalence of infection than wild ones^12,47,48^. Studies of terrestrial NHPs found a high STH prevalence of 81.2% in captive orangutans with ground dwelling behaviour^8,47,49,50^. In addition to these studies, our results significantly demonstrate that more semi‐wild orangutans were positive for *O. aculeatum* than wild orangutans. However, it should be noted that this may be because of a sampling bias or over‐representation of semi‐wild orangutan samples in the dataset. As the presence of nodular worms have been confirmed in Asian primates in this and further studies with larger sample sizes should be carried out to confirm these findings.

However, several behavioural and ecological factors may contribute to higher infection with nodular worm species among semi-captive primates, such as restricted ranges making it difficult to avoid contaminated areas, changes in social structure, increased population density, dietary changes and stress and more contact with other animal host species. Another significant factor could be decreased arboreality and increased terrestrial locomotion in semi-captive animals, which allows more contact with the soil and increases exposure to STHs infective larvae. Regarding wild NHPs in Asia, previous studies have reported that the percentage of wild orangutans infected by STHs were 33% for *P. pygmaeus*^8^ and 47% for *P. abelii* ^47^. In this study, we report that 30% of free ranging animals were infected with *O. aculeatum* alone. Apart from decreased arboreal life style and increased contact with soil, increasingly overlapping home ranges with other wildlife species and livestock may result in increased levels of infection and parasite occurrence^8,47,51^. Additionally, the habitat along the swamp forest seems to be an ideal environment for the development of soil-transmitted species as it provides the required humidity^7^.

In addition, Ghai et al.^28^ found that, in the case of *Oesophagostomum*, smaller primate groups with large daily travel distances had a higher prevalence. The free ranging orangutans have large home ranges, for example wild orangutans home ranges are estimated to be 423 ha/month for males and 131 ha/month for females^52^. However, in our study the free ranging animals with larger habitat ranges were not observed to have higher prevalence’s of infection than semi-wild animals. The highest prevalence of *O. aculeatum* in orangutans was found in Bukit Lawang (75%) despite it having the lowest orangutan density (1.8 individuals/km^2^)^48^, suggesting orangutan to orangutan transmission may not be a common source of infection directly or indirectly. Although Frias et al.^38^ suggested that orangutans may also acquire *Oesophagostomum* from other NHPs, in our study, this could be related to increased human activity in that area^48^ and increased approaching of humans, their dwellings and domestic animals or by semi-wild orangutans who have been previously exposed to human contact during rehabilitation. Ghai et al.^28^ observed that, some primates, particularly those traversing large distances in small groups, were most susceptible to nodule worm infection. If data were available to investigate the relationship between range size and prevalence in wild orangutans and gibbons, a similar pattern observed by Ghai et al.^28^ would be interesting to test.

The observed low infection prevalence from free ranging NHPs may also point towards the hypothesis that great apes are able to control parasite infection and avoid the progression of the disease by self-medication, i.e. leaf swallowing^35,53,54^ and wild animals have more access to these plants. Recently the first report of leaf swallowing in an Asian species, the white‐handed gibbon, suggested a similar self‐medicative function against nematode infection^55^.

In this study, the patterns of genetic variability observed among populations differ. A high level of genetic diversity was found across all three *O. aculeatum* populations. A significant difference in the number of polymorphic sites, nucleotide diversity and average number of nucleotide differences was evident between wild and semi-wild populations. Free ranging populations had higher genetic diversity than semi-wild ones. In wildlife, genetic diversity is probably greater than in anthropized environments, possibly due to a larger range of hosts^56,57^. Within free ranging populations, the Seb population had a higher genetic diversity then Suaq. This higher genetic diversity was due to observed unique haplotypes, geographical isolation, population size and different host species. Moreover, *O. aculeatum*, is a specialised organism that infects a specific host and the genetics and behaviour of a host can play an important role on the parasites genomic variation within the different host species (*P. pygmaeus*, *P. abelii* and *H. albibarbis*). Our assessment of population genetic differentiation calculating *Fst* index revealed non-significant differentiation among Sumatran populations and indicated low levels of genetic differentiation within the same host population. This pattern was also observed between the two free ranging populations, Seb and Suaq, from different host species. Interestingly our result showed significant genetic diversity between Buk and Seb populations. The isolation by distance analysis indicated that more genetically similar relationships were found for more distant populations then nearby populations (between Suaq and Seb then between two neighbour semi wild and wild Sumatran, Suaq and Buk population). Thus, *O. aculeatum* isolated from different geographical regions may differ because of a heterogeneous species, less population diffusion, complex genetic structure and high differentiation.

In our study, the molecular analysis was limited to a single gene. While several studies demonstrated a relatively high genetic diversity in the *Oesophagostomum* genus^27,28,46^, the sequencing of additional genes or other molecular markers should be applied to confirm species level differentiation and the population genetic structure of *O. aculeatum* in Southeast Asia.

The origin of *O. aculeatum* is unclear but it is clear that *O. aculeatum* is confined to Asia and infects more wild populations such as other primates and elephants^37,38,58^. Faecal examination of long-tailed macaques in Asia has uncovered *Oesophagostomum* spp. infections^59^. The Asian non-human primates including orangutans and gibbons, inhabiting the same area as elephants or other primates might provide evidence of *O. aculeatum* transmission between species from different environmental sources.

*O. bifurcum* and *O. stephanostomum* have been identified with certainty in reported *Oesophagostomum* human infections^33^, however, possible *O. aculeatum* infections have also been reported in humans in Southeast Asia^60–62^. To our knowledge, outbreaks of Oesophagostomosis in human populations have not been documented in Indonesia. Further work to test humans in the area and further genotyping would help to understand *O. aculeatum* infections in human.

Although other *Oesophagostomum* spp*.* such as *O. stephanostomum* and *O. bifurcum* are regarded as zoonotic^33^ the lack of studies characterising *O. aculeatum* in wildlife makes it different to understand the epidemiology of this species and further investigations are needed to tackle this problem. However, we do not reject the possibility of *O. aculeatum* having a zoonotic potential as already proposed by Frias et al.^38^.

Our results confirm that *O. aculeatum* should be considered a pathogen for non-human primates in Southeast Asia. *Oesophagostomum* in semi-wild animals were found at a higher prevalence but showed a lower genetic variability compared with the ones in fully wild animals, further demonstrating the influence of human activities on the parasite dynamics in wild primate species. Further studies should be conducted to better understand the epidemiology and dynamic of *Oesophagostomum* transmission between human, non-human primates, other wild species and livestock, in Southeast Asia, following a one-health approach. Of particular interest are changes in transmission and prevalence in the context of the increasing human perturbation of natural forested habitats in Southeast Asia.

## Materials and methods

### Study site, sample collection and origin of faecal samples

Faecal samples were collected from three sites in Indonesia, one site in Borneo and two sites in Sumatra between 2004 and 2011. In Borneo, 11 faecal samples from orangutans (*P. pygmaeus)* and 5 samples from gibbons (*H. albibarbis*) were collected in Sebangau. Samples were collected within the LAHG (Laboratorium Alam Hutan Gambut: The Natural Laboratory for the Study of Peat Swamp Forest), an area of 500 km^2^ in the north-east of the Sebangau Forest, which was designated for the purpose of scientific research in 1997, and is managed by CIMTROP (Centre for International Cooperation in Management of Tropical Peatland) at the University of Palangka Raya. Sebangau is located in Central Kalimantan and consists of peat swamp forest. 39 faecal samples from Sumatran orangutans (*P. abelii*) were collected from two localities in the Gunung Leuser National Park. Suaq Balimbing is a coastal swamp situated on the western border of Gunung Leuser National Park. Bukit Lawang is located in Northern Sumatra, on the eastern border of the Gunung Leuser National Park, in hill dipterocarp forest (Fig. 1 and Table 1).

Except for orangutans in Bukit Lawang, all animals in this study can be considered wild. They have no physical contact with humans. In Bukit Lawang orangutans are considered semi-wild because they were released after a reintroduction process and therefore are less hesitant to have contact with humans^12^.

Wild and semi-wild animals in the field were followed from nest to nest, for several hours and/or days. All faecal samples were collected immediately after defecation from identified known individuals in conformity with the ethical treatment of non‐human primates. Collected samples were preserved in ethanol (96%). Given codes, sex, animal category, population and location were recorded for each animal (Supp Table 1).

### Collection of eggs and microscopy

For each sample; two grams of faecal material was homogenised with 15 ml water and filtered thought a 100 µm sieve ( ~) for microscopic examination. The filtered material was then examined using Sheather’s flotation solution^63^ and the sedimentation method, recommended in the approved guidelines of the Clinical and Laboratory Standards Institute for identification of intestinal tract parasites^64^. The whole sediment sample examination was repeated several times after the same sedimentation. All observed eggs were examined under an Olympus BX50 light microscope under the magnification X100. Egg size, shape, colour and internal structures were recorded. Eggs tentatively identified as *Oesophagostomum* spp., based on morphological characteristics, were transferred using a micropipette to eppendorf tubes contain 200 μl PBS for molecular confirmation.

### DNA extraction, PCR conditions and sequencing

The isolated eggs from one individual were combined into one tube and were washed and centrifuged twice with 200 μl PBS to remove any residual ethanol. DNA was extracted using the QIAamp DNA mini kit. DNA was purified from tissues (Qiagen, France) according to the manufacturer’s recommendation with the following modifications: samples were subsequently mixed with 0.2 g sterile mixture of 0.1/0.5 mm glass beads (Bertin Technologies, www.precellys.com) and 180 μl of ATL buffer (Qiagen). Samples were homogenised using a TissueLyser (Qiagen Retsch GmbH, Hannover, Germany) for 5 min at maximum speed. Hereafter, the suspensions were incubated over night at 56 °C. DNA was eluted in 70 μl of distillate water.

The entire ITS1–5.8S–ITS2 region of the *Oesophagostomum* genus was amplified by a two-step semi nested PCR using forward and reverse primers (Supp Table 2) flanking the 3′ terminus of 18S rDNA and the 5′ terminus of 28S r DNA, respectively. PCR amplification was performed in 25 μl containing 12.5 μl AmpliTaq Gold Master Mix, 0.5 μl of 10 μM each primer and 5 μl of template. The second of the two nested PCR steps was done following the same protocol using 3 μl of the first PCR product as template. For each run, as a negative control, nuclease-free water was added to the PCR mix instead of the DNA sample.

The PCR I cycles were: 95 °C (10 min), [95 °C (30 s), 55 °C (30 s), 72 °C (1 min)] for 40 cycles and 7 min at 72 °C. The PCR IIa cycles were: 95 °C (10 min), [95 °C (30 s), 53 °C (30 s), 72 °C (45 s)] for 40 cycles and 7 min at 72 °C. The PCR IIb: 95 °C (10 min), [95 °C (30 s), 55 °C (30 s), 72 °C (45 s)] for 40 cycles and 7 min at 72 °C.

The amplified products were subjected to 2% agarose gel electrophoresis in TAE buffer. PCR products were commercially purified and sequenced in both directions using nested PCR primers by Macrogen (Macrogen, Amsterdam, Netherlands).

### Statistical analysis

Only PCR positive samples were taken for analysis. Statistical analysis was carried out using R version 3.5.1^65^. Generalized linear models are recommended for the analyses of parasite data but as full factorial or simpler models could not be fitted due to lack of convergence, χ2 tests were used. Tests carried out were two‐tailed with Yates correction. Animals were deemed positive if any faecal sample collected from them during the study tested positive by PCR. A p ≤ 0.05 was considered statistically significant. For more details, see Stuart et al^12^.

### Sequence alignment, sequences and phylogenetic analyses

All sequences were manually checked, assembled and concatenated using CodonCode Aligner software (www.codoncode.com). Sequences were aligned using Clustal W with Bioedit software version 7.2.5.

For each population, aligned FASTA files were collapsed into variable sites and haplotypes for parsimony network were reconstructed using DNASP v.5.10.01^66^. A statistical parsimony network to infer relationships among sequences was created with Network 5.0^67^.

To examine the relationship of the all known Oesophagostomum species to date, a phylogenetic tree was constructed using a set of reference sequences belonging to different species from Genbank (Supplementary Table S4). For a more descriptive tree, only two of the multiple representative sequences of a haplotype were used in analysis. Phylogenetic analyses were then performed after multiple alignments of the obtained partial ITS1–5.8S–ITS2 sequences (258 nucleotides). We used both Bayesian (Fig. 3) and maximum likelihood (ML) (Supplementary Figure S2) methods for phylogenetic tree construction. The best-fitting ML model under the Akaike information criterion was HKY86 + G6 for nucleotides as identified by JModelTest v.0.1.1^40^.

For phylogenetic Bayesian inferences, the software MrBayes 3.2.6_1^68^ was performed using the web service of Phylogeny.fr (https://ngphylogeny.fr/tools/tool/281/form). Markov Chain Monte Carlo (MCMC) parameters were set to sample a tree every 10 or 100 generations of 100.000 generations and the burn-in was set at 250 trees sampled. Resulted tree was accepted when the average standard deviation of split frequencies is lower than 0.05 and the average Potential Scale Reduction Factor (PSRF) for parameter values is around 1.0 (0.9–1.1), simultaneously.

The highest-likelihood DNA trees and corresponding bootstrap support values were obtained by PhyML^69^ (freely available at the ATGC bioinformatics platform http://www.atgc-montpellier.fr/) using nearest neighbour interchange plus subtree pruning recrafting (NNI + SPR) branch swapping and 100 bootstrap replicates. *Necator americanus* (LC036563) was used to root the tree. The resulting tree was drawn using iTOL v.5.4^70^.

### Genetic diversity and population differentiation

The genetic diversity estimates (N, Number of sequence obtained; S, Number of polymorphic sites; K, Number of haplotypes; Hd, Haplotype diversity; π, Nucleotide diversity; D, Average number of nucleotide difference between populations) were computed using DNASP v.5.10.01^66^. Finally, to evaluate whether the genetic differentiation between populations was associated with the geographical isolation of islands, analysis of molecular variance AMOVA was performed in Arlequin ver 3.1^71^. Mantel test for matrix correlation between genetic distance and geographic distance was performed by using IBD^72^ with 1000 permutations.

### Ethics approval

All the research reported in this manuscript adhered to the legal requirements of the country in which the work took place. Since the collection of faecal samples from orangutans and gibbons was non-invasive and did not involve interaction with or distress to the animals, the study was not reviewed by an animal ethics committee.

## Supplementary Information

Supplementary Information.

## Data Availability

The newly generated sequences were deposited in the GenBank database under the accession numbers: MW756966–MW756992.
