# Supplementary material for: Genetic characterization of nodular worm infections in Asian Apes
Source: Sci Rep. 2021 Mar 31;11:7226. doi: 10.1038/s41598-021-86518-2 (PMC8012698; doi:10.1038/s41598-021-86518-2)
Supplement: Supplementary file 1 — Supplementary Information. [file 41598_2021_86518_MOESM1_ESM.docx]

Supplementary Information

Genetic Characterization of Nodular Worm Infections in Asian apes.

Yalcindag et al.

Supplementary Table S1…………………………………………………………….2-4

Characteristics of the fecal samples collected and sequenced in this study.

Supplementary Table S2…………………………………………………………….5

Primers sequences of each gene for first and second PCR.

Supplementary Table S3………………………………………………………….....5

Mean of egg measurement between three populations.

Supplementary Table S4…………………………………………………………….6-8

List of all sequences used in this study.

Supplementary Figure S1……………………………………………………………9

Isolation by distance

Supplementary Figure S2……………………………………………………………10

Phylogenetic tree from Maximum Likelihood

References……………………………………………………………………………11

**Supplementary Table S1**: Characteristics of the orangutans and gibbons fecal samples collected and sequenced in this study with, individual codes, sex, animal category, population, locations and host species.

| **Given Codes** | **Sex** | **Animal Category** | **Population** | **Location** | **Host species** | **Analyse** | |  |
| --- | --- | --- | --- | --- | --- | --- | --- | --- |
|  |  |  |  |  |  | **microscopy** | **PCR** |  |
| Seb 7 | ♂ | W | Sebangau | Borneo | *P.p* |  |  | |
| Seb 10 | ♀ | W | Sebangau | Borneo | *P.p* |  | x | |
| Seb 48 | ♂ | W | Sebangau | Borneo | *H.a* | x | x | |
| Seb 96 | ♀ | W | Sebangau | Borneo | *P.p* | x |  | |
| Seb 98 | ♂ | W | Sebangau | Borneo | *P.p* | x | x | |
| Seb 100 | ♂ | W | Sebangau | Borneo | *P.p* | x | x | |
| Seb 102 | NA | W | Sebangau | Borneo | *H.a* | x | x | |
| Seb 123 | NA | W | Sebangau | Borneo | *H.a* |  |  | |
| Seb 151 | ♂ | W | Sebangau | Borneo | *P.p* | x |  | |
| Seb 235 | ♂ | W | Sebangau | Borneo | *H.a* | x | x | |
| Seb 264 | ♀ | W | Sebangau | Borneo | *P.p* | x |  | |
| Seb 267 | ♀ | W | Sebangau | Borneo | *H.a* |  |  | |
| Seb 289 | ♀ | W | Sebangau | Borneo | *P.p* | x |  | |
| Seb 348 | ♂ | W | Sebangau | Borneo | *P.p* |  |  | |
| Seb 350 | ♂ | W | Sebangau | Borneo | *P.p* |  |  | |
| Seb 375 | ♂ | W | Sebangau | Borneo | *P.p* |  |  | |
|  |  |  |  |  |  |  |  |  |
| Suaq 156 | ♀ | W | Suaq | Sumatra | *P.a* |  |  | |
| Suaq 157 | ♂ | W | Suaq | Sumatra | *P.a* |  |  | |
| Suaq 160 | ♀ | W | Suaq | Sumatra | *P.a* | x | x | |
| Suaq 161 | ♀ | W | Suaq | Sumatra | *P.a* | x |  | |
| Suaq 162 | ♂ | W | Suaq | Sumatra | *P.a* |  |  | |
| Suaq 163 | ♂ | W | Suaq | Sumatra | *P.a* |  |  | |
| Suaq 164 | ♂ | W | Suaq | Sumatra | *P.a* | x |  | |
| Suaq 167 | ♂ | W | Suaq | Sumatra | *P.a* |  |  | |
| Suaq 168 | ♂ | W | Suaq | Sumatra | *P.a* | x | x | |
| Suaq 169 | ♂ | W | Suaq | Sumatra | *P.a* |  |  | |
| Suaq 170 | ♂ | W | Suaq | Sumatra | *P.a* | x | x | |
| Suaq 171 | ♀ | W | Suaq | Sumatra | *P.a* |  |  | |
| Suaq 172 | ♂ | W | Suaq | Sumatra | *P.a* |  |  | |
| Suaq 173 | ♂ | W | Suaq | Sumatra | *P.a* |  |  | |
| Suaq 174 | ♂ | W | Suaq | Sumatra | *P.a* | x |  | |
| Suaq 175 | ♂ | W | Suaq | Sumatra | *P.a* | x | x | |
| Suaq 425 | ♀ | W | Suaq | Sumatra | *P.a* | x |  | |
| Suaq 426 | ♂ | W | Suaq | Sumatra | *P.a* | x | x | |
| Suaq 427 | ♀ | W | Suaq | Sumatra | *P.a* | x | x | |
|  |  |  |  |  |  |  |  |  |
| Buk 4 | ♀ | SW | Bukit Lawang | Sumatra | *P.a* | x |  | |
| Buk 6 | ♀ | SW | Bukit Lawang | Sumatra | *P.a* | x | x | |
| Buk 18 | ♀ | SW | Bukit Lawang | Sumatra | *P.a* | x | x | |
| Buk 31 | ♀ | SW | Bukit Lawang | Sumatra | *P.a* | x | x | |
| Buk 62 | ♀ | SW | Bukit Lawang | Sumatra | *P.a* | x | x | |
| Buk 67 | ♂ | SW | Bukit Lawang | Sumatra | *P.a* | x | x | |
| Buk 68 | ♂ | SW | Bukit Lawang | Sumatra | *P.a* | x | x | |
| Buk 70 | ♂ | SW | Bukit Lawang | Sumatra | *P.a* | x | x | |
| Buk 73 | ♀ | SW | Bukit Lawang | Sumatra | *P.a* | x | x | |
| Buk 74 | ♂ | SW | Bukit Lawang | Sumatra | *P.a* | x |  | |
| Buk 101 | ♂ | SW | Bukit Lawang | Sumatra | *P.a* | x | x | |
| Buk 104 | ♀ | SW | Bukit Lawang | Sumatra | *P.a* | x | x | |
| Buk 105 | ♀ | SW | Bukit Lawang | Sumatra | *P.a* | x | x | |
| Buk 106 | ♀ | SW | Bukit Lawang | Sumatra | *P.a* | x |  | |
| Buk 107 | ♀ | SW | Bukit Lawang | Sumatra | *P.a* | x |  | |
| Buk 188 | ♀ | SW | Bukit Lawang | Sumatra | *P.a* | x | x | |
| Buk 200 | ♂ | SW | Bukit Lawang | Sumatra | *P.a* |  |  | |
| Buk 218 | ♀ | SW | Bukit Lawang | Sumatra | *P.a* | x | x | |
| Buk 334 | ♂ | SW | Bukit Lawang | Sumatra | *P.a* |  | x | |
| Buk 405 | ♀ | SW | Bukit Lawang | Sumatra | *P.a* | x | x | |

♂: Male, ♀: Female, NA: Data not avaiable, W: wild animals, SW: semi-wild animals, *P.p*: *Pongo pygmaeus*, *P.a*: *Pongo abelii*, *H.a*: *Hylobates albibarbis*

**Supplementary Table S2:** Primers sequences of each gene for first and second PCR.

* Primers of second PCR used for sequencing these genes.

**Supplementary Table S3:** Mean of egg measurement between three populations.

| **Population** | **LONG (µm)** | **WIDE (µm)** |
| --- | --- | --- |
| Suaq | 70,11 | 42,36 |
| Sebangau | 69,96 | 42,71 |
| Bukit Lawang | 68,35 | 39,08 |

**Supplementary Table S4:** List of all sequences used in this study from Genbank.

| Genbank accession number | Organism | Host | Country | Reference |
| --- | --- | --- | --- | --- |
| AB586134 | *Oesophagostomum cf. aculeatum* | Japanese macaque | Japan | ^1^ |
| LC063721 | *Oesophagostomum aculeatum* | Japanese macaque | Japan | ^2^ |
| LC063722 | *Oesophagostomum aculeatum* | Japanese macaque | Japan |  |
| LC428824 | *Oesophagostomum aculeatum* | Bornean orangutan | Malaysia/Sabah | ^3^ |
| KF319022 | *Oesophagostomum aculeatum* | Long-tailed macaque | China | ds* |
| KF319023 | *Oesophagostomum aculeatum* | Long-tailed macaque | China |  |
| AB971665 | *Oesophagostomum asperum* | Goat | Japan | ^4^ |
| JN835419 | *Oesophagostomum asperum* | Cashmere goat | China | ^5^ |
| JN835420 | *Oesophagostomum asperum* | Cashmere goat | China |  |
| JX188461 | *Oesophagostomum asperum* | Goat | China | ^6^ |
| JX188457 | *Oesophagostomum asperum* | Goat | China |  |
| JX188459 | *Oesophagostomum asperum* | Goat | China |  |
| JX188464 | *Oesophagostomum asperum* | Goat | China |  |
| JX188465 | *Oesophagostomum asperum* | Goat | China |  |
| JX188467 | *Oesophagostomum asperum* | Goat | China |  |
| JX188469 | *Oesophagostomum asperum* | Goat | China |  |
| KM200790 | *Oesophagostomum asperum* | Goat | China | ^7^ |
| KM200795 | *Oesophagostomum asperum* | Goat | China |  |
| KM200786 | *Oesophagostomum asperum* | Goat | China |  |
| KM200803 | *Oesophagostomum asperum* | Goat | China |  |
| KM200805 | *Oesophagostomum asperum* | Goat | China |  |
| AF136575 | *Oesophagostomum bifurcum* | Mona monkey | NA | ^8^ |
| LC063694 | *Oesophagostomum bifurcum* | Eastern chimpanzee | Uganda | ^2^ |
| LC063695 | *Oesophagostomum bifurcum* | Eastern chimpanzee | Uganda |  |
| LC063712 | *Oesophagostomum bifurcum* | Yellow baboon | Tanzania |  |
| LC063716 | *Oesophagostomum bifurcum* | Yellow baboon | Tanzania |  |
| LC063717 | *Oesophagostomum bifurcum* | Yellow baboon | Tanzania |  |
| LC063720 | *Oesophagostomum bifurcum* | Chacma baboon | South Africa |  |
| LC063713 | *Oesophagostomum bifurcum* | Yellow baboon | Tanzania |  |
| LC063715 | *Oesophagostomum bifurcum* | Yellow baboon | Tanzania |  |
| KF319024 | *Oesophagostomum bifurcum* | Long-tailed macaque | China | ds* |
| KF319026 | *Oesophagostomum bifurcum* | Long-tailed macaque | China |  |
| KT215379 | *Oesophagostomum bifurcum* | Yellow baboon | Kenya | ^9^ |
| MT184891 | *Oesophagostomum bifurcum* | Olive baboon | Kenya | ds* |
| Y11733 | *Oesophagostomum bifurcum* | Human | NA | ^10^ |
| AJ006150 | *Oesophagostomum columbianum* | Sheep | China | ^11^ |
| JX188470 | *Oesophagostomum columbianum* | Sheep | China | ^6^ |
| JX188471 | *Oesophagostomum columbianum* | Sheep | China |  |
| JX188472 | *Oesophagostomum columbianum* | Sheep | China |  |
| JX188473 | *Oesophagostomum columbianum* | Sheep | China |  |
| JX188474 | *Oesophagostomum columbianum* | Sheep | China |  |
| JX188475 | *Oesophagostomum columbianum* | Sheep | China |  |
| MT653093 | *Oesophagostomum columbianum* | Goat | Bangladesh | ds* |
| AJ889569 | *Oesophagostomum dentatum* | Wild boar | China | ^12^ |
| AJ889570 | *Oesophagostomum dentatum* | Wild boar | China |  |
| AJ889571 | *Oesophagostomum dentatum* | Wild boar | China |  |
| AJ619979 | *Oesophagostomum dentatum* | Wild boar | China | ds* |
| KU891915 | *Oesophagostomum dentatum* | NA | Austria | ds* |
| KU891916 | *Oesophagostomum dentatum* | NA | Austria |  |
| KU891917 | *Oesophagostomum dentatum* | NA | Austria |  |
| Y11735 | *Oesophagostomum dentatum* | Pig | NA | ^13^ |
| JX159791 | *Oesophagostomum environmental sample* | Gorilla | Cameroon | ^14^ |
| JX159803 | *Oesophagostomum environmental sample* | Gorilla | Cameroon |  |
| JX159816 | *Oesophagostomum environmental sample* | Gorilla | Cameroon |  |
| JX159841 | *Oesophagostomum environmental sample* | Gorilla | Cameroon |  |
| JX159843 | *Oesophagostomum environmental sample* | Gorilla | Cameroon |  |
| JX159845 | *Oesophagostomum environmental sample* | Gorilla | Cameroon |  |
| AJ889568 | *Oesophagostomum quadrispinulatum* | Wild boar | China | ^12^ |
| Y11736 | *Oesophagostomum quadrispinulatum* | Pig | NA | ^13^ |
| AJ006149 | *Oesophagostomum radiatum* | NA | NA | ^11^ |
| KJ420893 | *Oesophagostomum radiatum* | Cattle | France | ^15^ |
| KJ420894 | *Oesophagostomum radiatum* | Cattle | France |  |
| KP150505 | *Oesophagostomum radiatum* | Cattle | USA | ^16^ |
| KP150518 | *Oesophagostomum radiatum* | Cattle | USA |  |
| MN833666 | *Oesophagostomum radiatum* | Cattle | Australia | ^17^ |
| MN833667 | *Oesophagostomum radiatum* | Cattle | Australia |  |
| MN833668 | *Oesophagostomum radiatum* | Cattle | Australia |  |
| KJ420900 | *Oesophagostomum sikae* | Roe deer | France | ^15^ |
| KJ420901 | *Oesophagostomum sikae* | Red deer | France |  |
| KJ420902 | *Oesophagostomum sikae* | Red deer | France |  |
| KJ420903 | *Oesophagostomum sikae* | Roe deer | France |  |
| KJ420904 | *Oesophagostomum sikae* | Roe deer | France |  |
| KJ420905 | *Oesophagostomum sikae* | Red deer | France |  |
| KJ420906 | *Oesophagostomum sikae* | Red deer | France |  |
| AB821013 | *Oesophagostomum stephanostomum* | Gorilla | Gabon | ^18^ |
| AB821015 | *Oesophagostomum stephanostomum* | Gorilla | Gabon |  |
| AB821016 | *Oesophagostomum stephanostomum* | Gorilla | Gabon |  |
| AB821020 | *Oesophagostomum stephanostomum* | Gorilla | Gabon |  |
| AB821025 | *Oesophagostomum stephanostomum* | Central chimpanzee | Gabon |  |
| AF136576 | *Oesophagostomum stephanostomum* | Central chimpanzee | NA | ^8^ |
| LC063706 | *Oesophagostomum stephanostomum* | Eastern chimpanzee | Uganda | ^2^ |
| LC063699 | *Oesophagostomum stephanostomum* | Eastern chimpanzee | Uganda |  |
| LC063708 | *Oesophagostomum stephanostomum* | Eastern chimpanzee | Uganda |  |
| LC063697 | *Oesophagostomum stephanostomum* | Eastern chimpanzee | Uganda |  |
| LC063698 | *Oesophagostomum stephanostomum* | Eastern chimpanzee | Uganda |  |
| KR149646 | *Oesophagostomum stephanostomum* | Eastern chimpanzee | Uganda | ^19^ |
| KR149647 | *Oesophagostomum stephanostomum* | Human | Uganda |  |
| MT184886 | *Oesophagostomum stephanostomum* | Red-tailed monkey | Kenya | ^20^ |
| MT184887 | *Oesophagostomum stephanostomum* | Red-tailed monkey | Kenya |  |
| MW040124 | *Oesophagostomum stephanostomum* | Bonobo | DRC | ds* |
| MW040125 | *Oesophagostomum stephanostomum* | Bonobo | DRC |  |
| MW040128 | *Oesophagostomum stephanostomum* | Bonobo | DRC |  |
| MW040136 | *Oesophagostomum stephanostomum* | Bonobo | DRC |  |
| AB908964 | *Oesophagostomum sp.* | Goat | Laos | ^21^ |
| AB908965 | *Oesophagostomum sp.* | Goat | Laos |  |
| AB908966 | *Oesophagostomum sp.* | Goat | Laos |  |
| HQ844232 | *Oesophagostomum sp.* | Sheep | China | ds* |
| MT294438 | *Oesophagostomum sp.* | Sheep | Thailand |  |
| HQ283349 | *Oesophagostomum venulosum* | Sheep | NA |  |
| KC998755 | *Oesophagostomum venulosum* | Sheep | New Zealand | ^22^ |
| KC998756 | *Oesophagostomum venulosum* | Sheep | New Zealand |  |
| KC998757 | *Oesophagostomum venulosum* | Sheep | New Zealand |  |
| KJ420907 | *Oesophagostomum venulosum* | Roe deer | France | ^15^ |
| KJ420908 | *Oesophagostomum venulosum* | Roe deer | France |  |
| KJ420909 | *Oesophagostomum venulosum* | Roe deer | France |  |
| KJ420910 | *Oesophagostomum venulosum* | Red deer | France |  |
| KJ420911 | *Oesophagostomum venulosum* | Red deer | France |  |
| MG651894 | *Oesophagostomum venulosum* | Barbary sheep | Tunisia | ds* |
| MG651895 | *Oesophagostomum venulosum* | Barbary sheep | Tunisia |  |
| MG651896 | *Oesophagostomum venulosum* | Barbary sheep | Tunisia |  |
| Y10790 | *Oesophagostomum venulosum* | Sheep | NA | ^11^ |
| LC036563 | *Necator americanus* | Human | Japan | ds* |

NA: information not available, ds*: unpublished sequences that directly submitted to NCBI.

**Supplementary Figure S1**: Isolation by distance


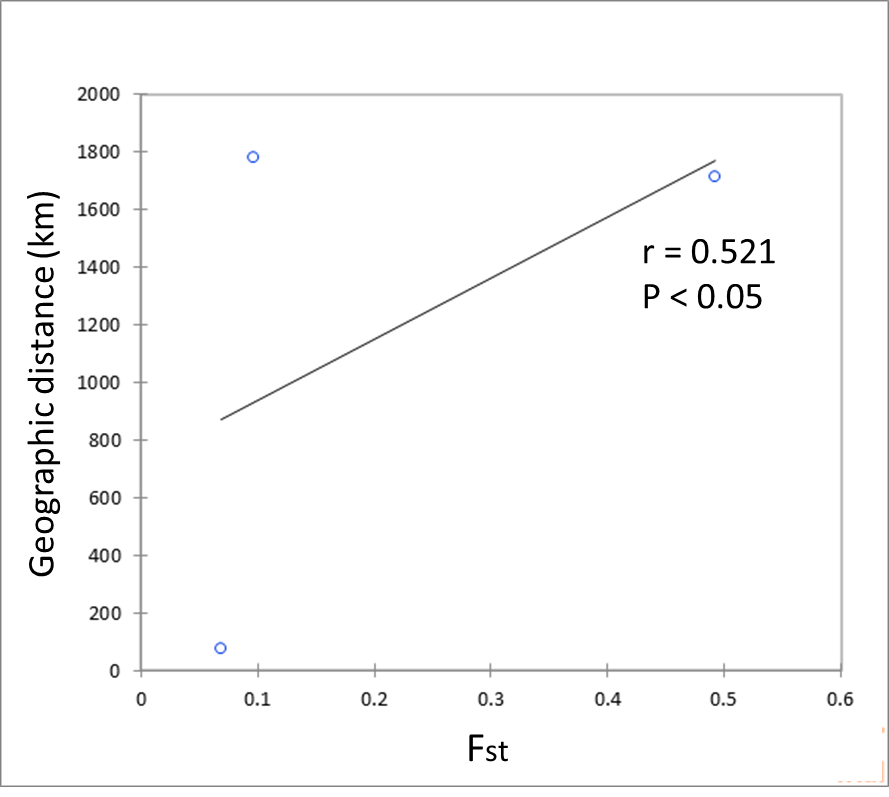


**Figure S1:** Isolation by distance (IBD) within each *O. aculeatum* population. Pairwise genetic differentiation (FST) plotted against pairwise geographic distances (km) along landmasses (r = 0.521; P = 0.5). No correlation between geographical distance and genetic distances.

**Supplementary Figure S2**: Phylogenetic tree from ML


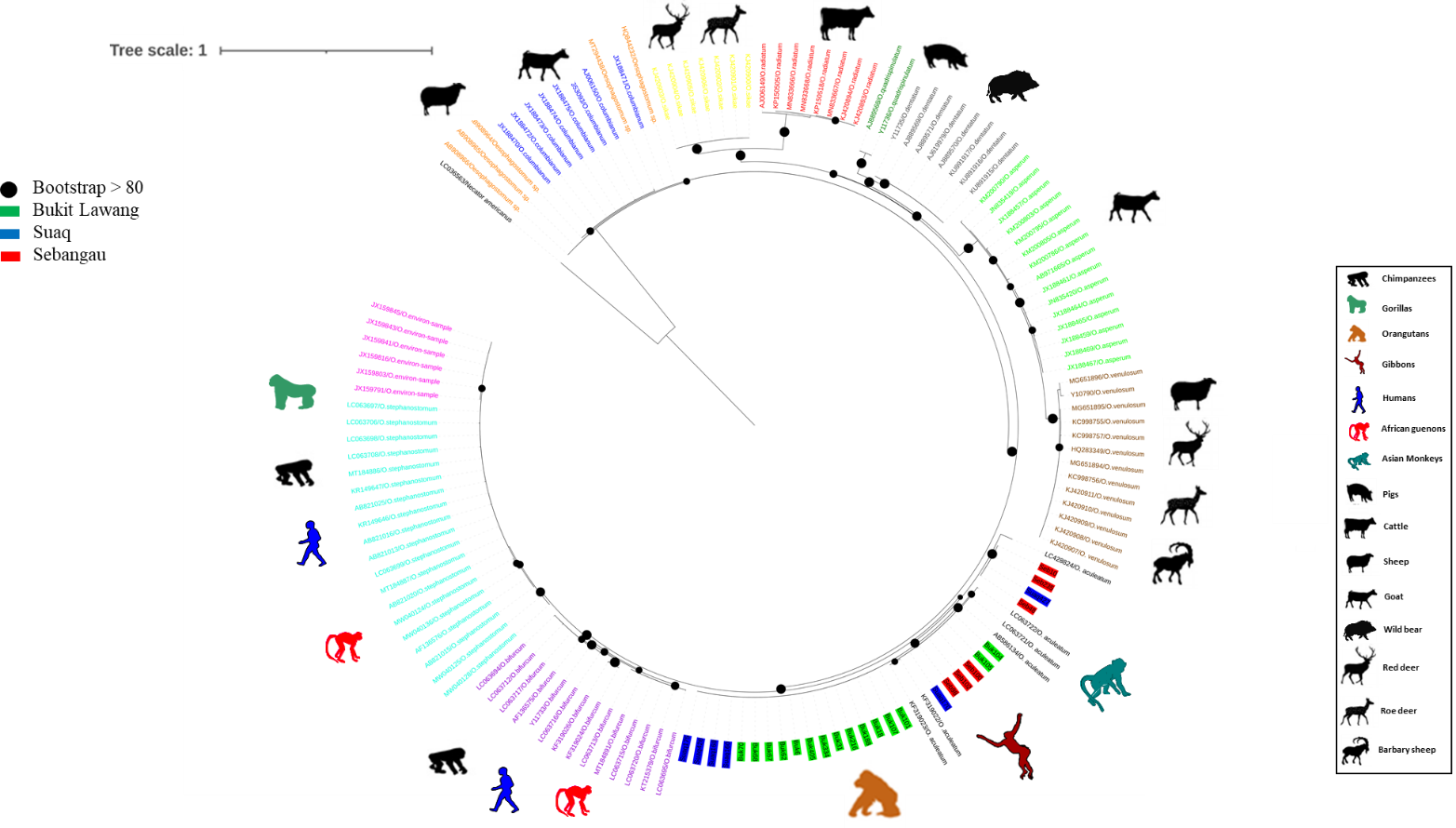


**Supplementary Figure S2**: Phylogenetic analysis of *Oesophagostomum* species based on ITS2 rDNA (258 bp) sequences (ML tree).

**References**

1 Arizono, N., Yamada, M., Tegoshi, T. & Onishi, K. Molecular Identification of Oesophagostomum and Trichuris Eggs Isolated from Wild Japanese Macaques. *Korean Journal of Parasitology* **50**, 253-257, doi:DOI 10.3347/kjp.2012.50.3.253 (2012).

2 Ota, N. *et al.* Molecular identification of Oesophagostomum spp. from 'village' chimpanzees in Uganda and their phylogenetic relationship with those of other primates. *R Soc Open Sci* **2**, 150471 (2015).

3 Frias, L. *et al.* Molecular characterization of nodule worm in a community of Bornean primates. *Ecol Evol* **9**, 3937-3945, doi:10.1002/ece3.5022 (2019).

4 Makouloutou, P., Matsuda, M., Haradono, K., Yanagida, T. & Sato, H. Oesophagostomum asperum infection in a domestic goat in Yamaguchi, Japan. *Jpn. J. Vet. Parasitol.* **13**, 16-20 (2014).

5 Yu, S.-K. *et al.* Phylogenetic studies of Oesophagostomum asperum from goats based on sequences of internal transcribed spacers of ribosomal deoxyribonucleic acid (DNA). *Afr J Microbiol Res* **6**, 3360-3365, doi:doi.org/10.5897/AJMR12.371 (2012).

6 Zhao, G. H. *et al.* Characterization of Oesophagostomum asperum and O. columbianum by internal transcribed spacers of nuclear ribosomal DNA. *J. Helminthol.* **88**, 74-81, doi:10.1017/S0022149X12000764 (2012).

7 Li, F. *et al.* Sequence variation in two mitochondrial DNA regions and internal transcribed spacer among isolates of the nematode Oesophagostomum asperum originating from goats in Hunan Province, China. *J. Helminthol.* **90**, 1-6, doi:doi.org/10.1017/S0022149X14000650 (2014).

8 Gasser, R. B., Woods, W. G., Huffman, M. A., Blotkamp, J. & Polderman, A. M. Molecular separation of Oesophagostomum stephanostomum and Oesophagostomum bifurcum (Nematoda: Strongyloidea) from non-human primates. *Int J Parasitol* **29**, 1087-1091, doi:10.1016/s0020-7519(99)00037-5 (1999).

9 Obanda, V. *et al.* Infection dynamics of gastrointestinal helminths in sympatric non-human primates, livestock and wild ruminants in Kenya. *PLoS One* **14**, e0217929, doi:10.1371/journal.pone.0217929 (2019).

10 Romstad, A. *et al.* Differentiation of Oesophagostomum bifurcum from Necator americanus by PCR using genetic markers in spacer ribosomal DNA. *Mol Cell Probes* **11**, 169-176, doi:10.1006/mcpr.1996.0094 (1997).

11 Newton, L. A., Chilton, N. B., Beveridge, I. & Gasser, R. B. Systematic relationships of some members of the genera Oesophagostomum and Chabertia (Nematoda: Chabertiidae) based on ribosomal DNA sequence data. *Int J Parasitol* **28**, 1781-1789, doi:10.1016/s0020-7519(98)00144-1 (1998).

12 Lin, R. Q. *et al.* Characterization of Oesophagostomum spp. from pigs in China by PCR-based approaches using genetic markers in the internal transcribed spacers of ribosomal DNA. *Parasitol Res* **101**, 351-356, doi:10.1007/s00436-007-0498-7 (2007).

13 Newton, L. A. *et al.* Rapid PCR-based delineation of the porcine nodular worms, Oesophagostomum dentatum and O. quadrispinulatum. *Mol Cell Probes* **11**, 149-153, doi:10.1006/mcpr.1997.0097 (1997).

14 Keita, M. B., Hamad, I. & Bittar, F. Looking in apes as a source of human pathogens. *Microb Pathog* **77**, 149-154, doi:10.1016/j.micpath.2014.09.003 (2014).

15 Patrelle, C., Ferte, H. & Jouet, D. Identification of Chabertiidae (Nematoda, Strongylida) by PCR-RFLP based method: a new diagnostic tool for cross transmission investigation between domestic and wild ruminants in France. *Infect Genet Evol* **28**, 15-20 (2014).

16 Avramenko, R. W. *et al.* Exploring the Gastrointestinal "Nemabiome": Deep Amplicon Sequencing to Quantify the Species Composition of Parasitic Nematode Communities. *PLoS One* **10**, e0143559, doi:10.1371/journal.pone.0143559 (2015).

17 Francis, E. K., McKay-Demeler, J., Calvani, N. E. D., McDonell, D. & Slapeta, J. Which larvae are they? Use of single larva for the molecular confirmation of Cooperia pectinata and Cooperia punctata in Australian cattle. *Vet Parasitol* **278**, 109033, doi:10.1016/j.vetpar.2020.109033 (2020).

18 Makouloutou, P. *et al.* Prevalence and genetic diversity of Oesophagostomum stephanostomum in wild lowland gorillas at Moukalaba-Doudou National Park, Gabon. *Helminthologia* **51**, 83-93, doi:DOI 10.2478/s11687-014-0214-y (2014).

19 Cibot, M. *et al.* Nodular Worm Infections in Wild Non-human Primates and Humans Living in the Sebitoli Area (Kibale National Park, Uganda): Do High Spatial Proximity Favor Zoonotic Transmission? *PLoS Negl Trop Dis* **9**, e0004133 (2015).

20 Mbuthia, P. *et al.* Potentially zoonotic gastrointestinal nematodes co-infecting free ranging non-human primates in Kenyan urban centres. *Vet Med Sci*, doi:10.1002/vms3.424 (2021).

21 Sato, M. O. *et al.* ematode infection among ruminants in monsoon climate (Ban-Lahanam, Lao PDR) and its role as food-borne zoonosis. *Rev Bras Parasitol Vet 23* **23**, 80-84, doi:DOI:10.1590/S1984-29612014011 (2014).

22 Bisset, S. A., Knight, J. S. & Bouchet, C. L. A multiplex PCR-based method to identify strongylid parasite larvae recovered from ovine faecal cultures and/or pasture samples. *Vet Parasitol* **200**, 117-127, doi:10.1016/j.vetpar.2013.12.002 (2014).
